# Supplementary material for: Health and economic burden of insufficient physical activity in Saudi Arabia
Source: PLoS One. 2024 Apr 10;19(4):e0297278. doi: 10.1371/journal.pone.0297278 (PMC11006127; doi:10.1371/journal.pone.0297278)
Supplement: Attachment — Submitted filename: Response to reviewers.docx [file pone.0297278.s001.docx]

Response to Reviewers

When submitting your revision, we need you to address these additional requirements.

1. Please ensure that your manuscript meets PLOS ONE's style requirements, including those for file naming.

**Response: Manuscript was rechecked and aligned with the PLOS ONE's style requirements, including those for file naming.**

2. In your Data Availability statement, you have not specified where the minimal data set underlying the results described in your manuscript can be found. PLOS defines a study's minimal data set as the underlying data used to reach the conclusions drawn in the manuscript and any additional data required to replicate the reported study findings in their entirety. All PLOS journals require that the minimal data set be made fully available. For more information about our data policy, please see http://journals.plos.org/plosone/s/data-availability.

Upon re-submitting your revised manuscript, please upload your study’s minimal underlying data set as either Supporting Information files or to a stable, public repository and include the relevant URLs, DOIs, or accession numbers within your revised cover letter. For a list of acceptable repositories, please see http://journals.plos.org/plosone/s/data-availability#loc-recommended-repositories. Any potentially identifying patient information must be fully anonymized.

Important: If there are ethical or legal restrictions to sharing your data publicly, please explain these restrictions in detail. Please see our guidelines for more information on what we consider unacceptable restrictions to publicly sharing data: http://journals.plos.org/plosone/s/data-availability#loc-unacceptable-data-access-restrictions. Note that it is not acceptable for the authors to be the sole named individuals responsible for ensuring data access.

We will update your Data Availability statement to reflect the information you provide in your cover letter.

**Response: We have updated the manuscript with a statement regarding data availability and have provided a URL to a Github link that contains all of our input data and code.**

**https://github.com/Disease-Control-Priorities/PA_KSA**

Review Comments to the Author

Reviewer #1: The authors of this study investigated the health and economic burden of insufficient physical activity in Saudi Arabia, a country located in the Middle East region where most of the countries located in this region suffer from this public health issue. The study also benefits from a projection of the findings to 2040 and the expected burden is estimated in three scenarios of baseline, intervention, and ideal which could be interesting for future public health policy making in this country. Overall, the study benefits from a robust design and statistical analysis, and the manuscript is well drafted. However, some parts of the manuscript need some revisions to enhance the quality of material, with most of the comments on the methods section. Below, please find my comments and suggestions in this regard.

1. Methods: the two major parts of the methods section including “Estimating Avoidable Health Loss” and “Estimating Economic Impact” which are the most important parts of this paper are drafted inadequately in description and authors only mentioned they have used previously developed methods. Specifically, the last part on the economic burden estimation is too incomplete and need further expansion to make this research a valid and reliable one. In this regard, the incorporated models to estimate the economic burden should be fully elaborated in this section.

**Response: Thank you for this feedback. We have expanded the methods section to include additional technical details. We have also included a link to a Github page that contains all of our input data and code. Please also note that we made some minor improvements to our input data and analysis, with slightly different results. The main conclusions of the paper are unchanged.**

**We now emphasize the Potential Impact Fraction (PIF) rather than the Population Attributable Fraction (PAF) in the methods and results section. While PAFs can be calculated based on the input data, we think that reporting PIFs is more informative to the reader, since these are what are used to calculate the health and economic losses in our model. We have updated Fig 2 accordingly.**

2. Methods: the references for the pooled and used relative risks (RRs) to calculate the PAFs is not mentioned in this section which needs a revision to make everything is clear. Also, adding a table as a supplementary and providing the values of used RRs in this study is essential.

**Response: The revised manuscript now contains a citation for the relative risks we used. The RRs and other input data for calculating impact are provided on the Github page referenced above. We note the following statement in our original submission,**

***Relative risk estimates for colorectal cancer, breast cancer, ischemic heart disease, ischemic stroke, and type 2 diabetes mellitus for males and females aged 25-79 years were taken from other GBD studies [3, 13].***

**These are the RRs used in our analysis and reported on the Github page.**

3. Results: the presented section on findings lack appropriate tables including the estimated numbers of health and economic burden in different stratifications of population characteristics and according to the four health conditions. Therefore, enriching this section with informative tables is highly needed.

**Response: We have expanded Table 1 to include disaggregated results by age and sex, which are the most important population characteristics in our view.**

4. Limitations: one of the limitations which is not mentioned in the last part of the discussion is that authors have made their selection of the four conditions with highest burden linked to insufficient physical activity based on the GBD estimations and this could be biased as they secondarily use another data. So, mentioning this issue is highly suggested.

**Response: Thank you for highlighting this limitation. We have added the following to the limitations section on page 16: “Additionally, in order to ensure a focused and attainable research endeavor, we had to limit the scope exclusively to the four conditions exhibiting the highest burden associated with insufficient physical activity, as identified through the GBD estimates.”**

Reviewer #2:

1- Be consistent in using abbreviations. You should define abbreviations in their first use. Moreover, if you have defined any abbreviations, you should only use them instead of full terms (e.g., you used PA and “physical activity” many times interchangeably)

**Response: Thank you for raising this issue. We have proofread the paper and made all words/abbreviations consistent. We used physical activity not PA abbreviation.**

2- In the last paragraph of introduction, you should use present or future verbs. This should explain the main aim of your study.

**Response: Thank you, this has been edited - present verbs have been used.**

3- Add your study strengths prior to the limitations section.

**Response: Thank you for this suggestion, we have now added the strengths of the study on page 15, as follows: “This study is the first to present critical findings on the cost of physical inactivity in Saudi Arabia. It enhances our understanding of the economic repercussions of insufficient physical activity in the country, addressing a substantial public health issue and providing a case for investment. The study offers a thorough assessment using both mortality and DALYs. Finally, by translating health improvements into economic returns through standard cost-benefit analysis methods, the study provides a compelling argument for investment and practical insights for policymaking that can advance the physical activity policy agenda in Saudi Arabia.”**

4- Revise the whole manuscript for typos and grammatical errors.

**Response: We have proofread the manuscript, thank you for the guidance.**
